# LC-MS based metabolomics identification of natural metabolites against *Fusarium oxysporum*

**DOI:** 10.3389/fpls.2024.1435963

**Published:** 2024-09-03

**Authors:** Wenjuan Yang, Sidi Tang, Rubing Xu, Lu Zhang, Zihao Zhou, Yong Yang, Yanyan Li, Haibo Xiang

**Affiliations:** ^1^ State Key Laboratory of Biocatalysis and Enzyme Engineering, College of Life Sciences, Hubei University, Wuhan, China; ^2^ Tobacco Research Institute of Hubei Province, Wuhan, China

**Keywords:** *Fusarium oxysporum*, tobacco fusarium wilt, metabolomics, phenylpropiolic acid, linustatin, scopoletin

## Abstract

*Fusarium* is a soil-borne pathogen that poses a serious threat to the quality and yield of hundreds of crops worldwide, particularly tobacco production. Using metabolomics technology, we investigated natural metabolites from disease-conducting soil (DCS) and disease-suppressing soil (DSS) of tobacco rhizosphere as fungicides to control tobacco Fusarium wilt (TFW), which is mainly caused by *Fusarium oxysporum*. Furthermore, the antifungal mechanisms of these natural metabolites were preliminarily elucidated through various assessments, including antifungal activity determination, chemotaxis effect tests, PI staining experiments, and measurements of extracellular conductivity and protein content. Metabolomics results showed that the DCS with three different disease grades (G1, G5 and G9 groups) had significantly higher levels of 15, 14 and 233 differential rhizosphere metabolites (DRMs) and significantly lower levels of 72, 152 and 170 DRMs compared to the DSS (G0 group). According to KEGG pathway analysis, these DRMs were found to be enriched in the caffeine metabolism, biosynthesis of phenylpropanoids, galactose metabolism and tyrosine metabolism, etc. Linustatin, scopoletin and phenylpropiolic acid were picked out from these DRMs and found to have suppressive activity against *F. oxysporum* through correlation analysis and antifungal experiments. The three DRMs showed strong inhibitory effects on the growth and spore germination of *F. oxysporum* at concentrations of 0.5 mM or higher in each test period. Furthermore, *F. oxysporum* showed a phobotaxis effect against these three DRMs at concentrations as low as 0.25 mM. Finally, we found that the three DRMs had an inhibitory effect on *F. oxysporum* by destroying the integrity of the cell membrane and increasing the membrane permeability of *F. oxysporum*. This study firstly reports the inhibition activity of phenylpropiolic acid and linustatin on *F. oxysporum*, providing a practical and environmentally friendly method for biocontrol of TFW by using natural fungicides.

## 1 Introduction

*Fusarium* is a widespread soil-borne fungal pathogen that can infect hundreds of crops as diverse as tobacco, tomato, cotton, rice and banana, causing root rot, stem rot, or ear rot (Ma et al., 2013). It is commonly found in tobacco growing areas such as Hubei, Yunnan, Henan, Guizhou and Anhui Province in China and causes tobacco fusarium wilt (TFW) (Zhao et al., 2023). Infected tobacco plants show yellowing and wilting of the leaves, epidermal root rot, susceptibility to breakage and abscission, and browning of the vascular bundles. In severe cases, rot and necrosis occur from the stem base to the main root, with the xylem turning brown and black, and even leading to the death of the entire plant, which has a major impact on the yield and quality of the tobacco, becoming an inhibiting factor for the development of tobacco production.

To date, eleven species of TFW pathogens have been reported, including *Fusarium brachygibbosum*, *Fusarium commune*, *Fusarium equiseti, Fusarium falciforme*, *Fusarium fujikuroi*, *Fusarium kyushuense*, *Fusarium meridionale*, *Fusarium oxysporum*, *Fusarium sinensis*, *Fusarium solani*, and *Fusarium verticillioides*, in which *F. oxysporum* and *F. solani* were the main perpetrators in China (Fall et al., 2019; Gai et al., 2023; Li et al., 2023a; Rodríguez-Molina et al., 2007; Wang et al., 2013b; Zhong et al., 2020; Qiu et al., 2021a, b, 2022, 2023; Shen et al., 2023; Zhao et al., 2023). Currently, studies on the pathogenic mechanism of TFW mainly focus on *F. oxysporum*. It is well-known that *F. oxysporum* secretes various cell wall degrading enzymes and effectors to penetrate the roots and damage host plant defense systems, and then invade the root cortex and colonizes in the xylem vessels. The massively proliferating mycelium and small conidia in the vessels as well as the degradation products of the inner wall of the host vessel can block the vascular bundle, and disrupt water absorption and nutrient transport (Michielse and Rep, 2009; Gordon, 2017). On the other hand, *F. oxysporum* can produce various toxins such as fusaric acid, fumonisins, trichothecenes, zearalenone and ergosterol, which disrupt the normal physiological metabolism of host plants, and ultimately lead to plant wilting and death (Fernandes and Ghag, 2022).

The use of a TFW-resistant variety has been shown to be an effective and cost-effective method for controlling TFW (Shull et al., 2020). García (2015) developed a TFW-resistant variety named “Burley Pinar 2010” through five generations of self-pollination and lineage selection. In soils infected with Fusarium wilt, the yield of “Burley Pinar 2010” exceeded that of a normal variety. However, breeding a new TFW-resistant variety requires significant investment and time, which complicates the breeding work. Chemical management is currently the main TFW control method due to its high efficiency, convenience and economy. The use of chemical fungicides such as phenamacril (Zheng et al., 2022), prochloraz (Peng et al., 2022), and chloropicrin (LaMondia, 2015), leads to a significant reduction in Fusarium wilt, however, long-term use of these fungicides leads to the development of fungicide resistance of the pathogen and increases this difficulty to control (Zheng et al., 2022). In addition, inappropriate and excessive application can cause chemical residues, soil infertility and environmental pollution (Zhao et al., 2023).

Biocontrol of TFW using microorganisms and fungicides derived from plants or produced by microorganisms has become a hot topic due to its advantages in terms of effectiveness, biodegradability and environmental sustainability (Abbas and Yli-Mattila, 2022). For example, *Bacillus velezensis* Ba-0321 had an inhibitory effect of 75% on *F. oxysporum* and resulted in a high control effect on TFW of 81% (Li et al., 2023b). The natural product osthol separated from *Cnidium monnieri* (L.) Cusson can significantly disrupt the cell wall integrity and dynamic balance of *F. oxysporum* and has significant potential as an agent to control TFW (Zheng et al., 2021). The volatile organic compounds 2-methoxy-4-vinylphenol and 3,4-dimethoxystyrene, produced by the endophytic fungi *Sarocladium brachiariae* HND5, cause cell death by damaging the plasma membranes of *F. oxysporum* f. sp. cubense (Yang et al., 2021). These results indicate the potential of natural products as biocontrol agents against TFW. However, biopesticides are rarely registered for use against TFW, especially in China (Zhao et al., 2023). The main reason for this is that no biopesticides as effective as chemical fungicides have been developed to control TFW. Otherwise, they are more expensive and their application is more complicated than that of chemicals, especially in large-scale field cultivation (Egbuna et al., 2020). Therefore, it is crucial to find an effective and cost-effective biopesticide to control TFW.

The plant rhizosphere is a soil microecosystem composed of plants, soils, microbiomes and pathogens surrounding plant roots, and the essence of disease suppressing soil (DSS) is the balance of the soil microecosystem (Lv et al., 2023). Many studies focused on converting disease-conducting soil (DCS) into DSS by screening and employing beneficial microbes such as *Pseudomonas* (Raaijmakers and Weller, 1998), *Streptomycete* (Cha et al., 2016), *Trichoderma* (Tao et al., 2023), and obligate phages of pathogenic bacteria (Wang et al., 2019), etc. In particular, the discovery and the assembly of core microorganisms has become a focus [33], while the utilization of natural products with antipathogenic effects in the rhizosphere has been neglected. The aim of this study is to identify new metabolites negatively associated with TWB by comparing the different metabolites between DSS and DCS using metabolomics to provide a new source for the development of biopesticides to control TFW.

## 2 Materials and methods

### 2.1 Strain and chemicals

The pathogen *F. oxysporum* was provided by the Hubei Academy of Tobacco Sciences and cultured on PDA medium (200 g/L potato peels, 20 g/L dextrose, 15 g/L agar, pH 7.0) at 28°C. Alternariol, dibenzoyl thiamine, phenylpropiolic acid, ouabain, rosmarinic acid, linustatin, N-acetyl-Asp-Glu, estrone sulfate, scopoletin, D-(+)-glucose, acetaminophen glucuronide, pantothenic acid, 3-hydroxy-3-methylpentanedioic acid and propidium iodide were purchased from Aladdin Co. Ltd., China. All reagents were of analytical quality.

### 2.2 Rhizosphere soil collection

Tobacco cultivar Chuxue 26 was planted in a tobacco field continuously cultivated for 5 years in Xijiadian County (111.18354°E, 32.748336°N), Danjiangkou City, Hubei Province, China. At harvest time, tobacco plants were examined for disease severity and classified into grades 0-9 according to the GB/T23222-2008 “Classification and Investigation Methods for Tobacco Diseases and Pests” standard (Ren et al., 2008). Briefly, “G0” represents the plants without symptoms; “G1” represents the plant growth is generally normal or slightly stunted, with a few roots necrotic and appearing black, and the middle and lower leaves are chlorotic (or discoloration); “G5” represents the height of the diseased plant is one-third to one-half shorter than that of the healthy plant. Most of the roots are necrotic and black, and more than two-thirds of the leaves are wilted, with obvious dry tips and edges; and “G9” represents the dead plant. The TFW grade 0 rhizosphere soil was defined as DSS, and TFW grades 1, 5, and 9 were defined as DCS. Total rhizosphere soil was collected by five-point sampling methods. At least six plants were collected for each TFW class. All samples were transported to the laboratory immediately after collection and stored at −80°C for further analysis.

### 2.3 Untargeted metabolomics and data processing

Five grams of soil sample were resuspended with 100 mL 80% methanol by well vortexing for 10 min and then sonicated at 40 kHz for 30 min at 4°C. The samples were incubated on ice for 5 min and then centrifuged at 15,000 g at 4°C for 20 min. The supernatant was concentrated to 10 mL and 20 μL of which was injected into the LC-MS/MS system. UHPLC-MS/MS analyses were performed using a Vanquish UHPLC system (ThermoFisher, USA) coupled with an Orbitrap Q ExactiveTM HF-X mass spectrometer (ThermoFisher, USA) in Novogene Co., Ltd. (Beijing, China). The samples were injected onto a Hypersil Gold column (100 × 2.1 mm, 1.9 μm) with a 12-minute linear gradient and a flow rate of 0.2 mL/min. The mobile phases consisted of water with 0.1% formic acid (solvent A) and methanol (solvent B) for the positive polarity mode, and 5 mM ammonium acetate (pH 9.0, solvent A) and methanol (solvent B) for the mode with negative polarity. The solvent gradient was adjusted as follows: 2% B, 1.5 min; 2-85% B, 3 min; 85-100% B, 10 min; 100-2% B, 10.1 min; 2% B, 12 min. The Q Exactive™ HF-X mass spectrometer was operated in positive/negative polarity mode with scan range of 100-1500 (m/z), a spray voltage of 3.5 kV, a capillary temperature of 320°C, a sheath gas flow rate of 35 psi and auxiliary gas flow rate of 10 L/min as well as an S-lens operated RF level of 60, temperature of the additional gas heater of 350°C, and full scan with data-dependent MS/MS.

The raw data files generated by UHPLC-MS/MS were processed using the Compound Discoverer 3.3 (CD3.3, ThermoFisher) to perform peak alignment, peak picking, and quantitation for each metabolite. The main parameters were set as follows: peak area was corrected with the first QC, actual mass tolerance, 5ppm; retention time tolerance, 0.2 min; signal intensity tolerance, 30%; signal-to-noise ratio, 3; and minimum intensity, 10,0000. After that, peak intensities were normalized to the total spectral intensity. The normalized data was used to predict the molecular formula based on additive ions, molecular ion peaks and fragment ions. And then peaks were matched with the mzCloud, mzVault and MassList database to obtain the accurate qualitative and relative quantitative results. Statistical analyses were performed using the statistical software R (R version R-3.4.3), Python (Python 2.7.6 version) and CentOS (CentOS release 6.6), When data were not normally distributed, standardize according to the formula: sample raw quantitation value/(The sum of sample metabolite quantitation value/The sum of QC1 sample metabolite quantitation value) to obtain relative peak areas; and compounds whose CVs of relative peak areas in QC samples were greater than 30% were removed, and finally the metabolites’ identification and relative quantification results were obtained.

The identified metabolites were annotated using the KEGG database (https://www.genome.jp/kegg/pathway.html), the HMDB database (https://hmdb.ca/metabolites) and the LIPID Maps database (http://www.lipidmaps.org/). Partial least squares discriminant analysis (PLS-DA) was performed on metaX. Significantly differential rhizosphere metabolites (DRMs) were determined by a combination of univariate and multivariate analysis (fold change≥ 2 or ≤ 0.5, *P*< 0.05, VIP > 1) using R statistical software (version 3.4.3). The Venn diagram was created using Venny2.1 (https://bioinfogp.cnb.csic.es/tools/venny/). Volcano plots and heatmaps of DRMs were plotted by ggplot2 and pheatmap package in the R language. The functions of these metabolites and metabolic pathways were investigated using the KEGG database.

### 2.4 *In vitro* assay for antimicrobial effect of DRMs

The inhibitory effect of DRMs on the growth of *F. oxysporum* mycelium was determined using the method of Stringlis et al. (2018). Briefly, a 5-mm mycelial plug of a freshly produced fungal strain was inoculated into the center of the PDA plate containing 0.5 mM DRMs for 6 d in the dark at 28 °C. PDA plates supplemented with DMSO and 0.2 g·L^-1^ Delvocid were used as negative and positive controls, respectively. A microplate assay was used to quantify the effect of DRMs on the growth of *F. oxysporum.* First, 96-well plates (Corning, #3599) were filled with 100 μL of liquid potato dextrose broth (PDB) containing 5000 fungal spores for each well. Then 100 μL of PDB with different concentrations of DRM dissolved in 80% methanol was added to the wells and equal amounts of methanol was added as a blank control. The antibiotic Delvocid (0.3 g·L^-1^; DSM) was used as a positive control. The 96-well plates were incubated at 28°C for 6 d and fungal growth (OD_600_) was measured every 2 d.

### 2.5 Chemotaxis assay

The quantitative measurement of the chemotaxis of the response of *F. oxysporum* to the DRMs was performed based on the Turrà’s method (Turrà et al., 2015). Four milliliters of water agar (0.5%, w/v) containing freshly produced microconidia (2.5 × 10^6^ mL^-1^) were spread in a 90-mm diameter Petri dish. Three parallel lines, 5 mm apart, were marked in the center of the bottom of the Petri dish. Two square wells were cut in agar along the two marked lines, and then 50 μL of DRMs (0.25, 0.5 and 1 mM) or the solvent DMSO were added to each well. Pectin (1%, w/v) and its solvent H_2_O were used as positive and negative controls, respectively. The plates were incubated in the dark at 28°C for 12 h. The number of hyphal tips of germinating conidia pointing to the test compound, to the control, or to neither (neutral) was counted with a Zeiss microscope (200 × magnification, Axioimager). Chemotaxis was calculated as the percentage of the number of hyphae growing toward the test compound or towards the solvent. A total of 500 hyphal tips were counted for each test.

### 2.6 Determination of mycelial cell membrane integrity and permeability

Propidium iodide (PI) stains were used to determine cell membrane integrity of *F. oxysporum* according to Hu (Hu et al., 2023) with some modifications. A fresh mycelia plug was collected from the plate edge of a 7-day-old *F. oxysporum*, inoculated into PDB medium with different DRM concentrations, and cultured for 12 h (28°C, 180 r/min). The mycelia were centrifuged 5 000×g, 4°C for 5 min and washed three times with PBS buffer (50 mM, pH 7.4), followed by staining with 7.5 μM PI at 37°C for 20 min. The mycelium was then washed three times with PBS buffer, mounted on a slide, and observed under a fluorescence microscope (Zeiss LSM710, Germany) at an excitation wavelength of 535 nm and an emission wavelength of 590 nm.

The degree of damage to cell membrane permeability of *F. oxysporum* after DRM treatment was assessed by measuring extracellular relative conductivity and changes in protein content in the supernatant, as described by Hu (Hu et al., 2023). Fresh mycelia plugs were cultured in PDB medium. After 2 d, the mycelia were filtered with sterile gauze and rinsed with PBS, and then 0.5 g of mycelia were incubated in sterile water containing different concentrations of DRM (0.25, 0.5, 1 mM) for 24, 48 and 72 h, respectively. The supernatant obtained by centrifugation at 12,000 rpm for 10 min at 4°C was used to determine the conductivity with a DDSJ-318 conductivity meter (INESA, China) and the final conductivity was determined after 72 h by treating the mycelia in boiling water bath for 10 min. The relative conductivity was calculated using the following equation:


Relative electrical conductivity (REC,%)=[(R1−R0)/(R2−R0)]×100

Where R0: the initial conductivity at 0 h, R1: the conductivity at 24, 48, and 72 h, R2: the conductivity of dead treatment at 72 h.

In addition, the mycelium was treated as described above, and 5 mL of supernatant was used to measure protein concentrations using a BCA protein assay kit (ThermoFisher, USA).

### 2.7 Statistical analysis

All data were analyzed using the one-way analysis of variance (ANOVA) method with SPSS 22.0 software (SPSS Inc., Chicago, USA) and Duncan’s multiple range test was used to test the significance of the difference between treatments (*P*<0.05). The correlation between the relative abundance of DRMs and TFW grade were analyzed by cor () in R language (method=spearman). All the experiments were repeated at least three times.

## 3 Results

### 3.1 Changes in the metabolome and functional analysis of DRMs

**Figure 1:**
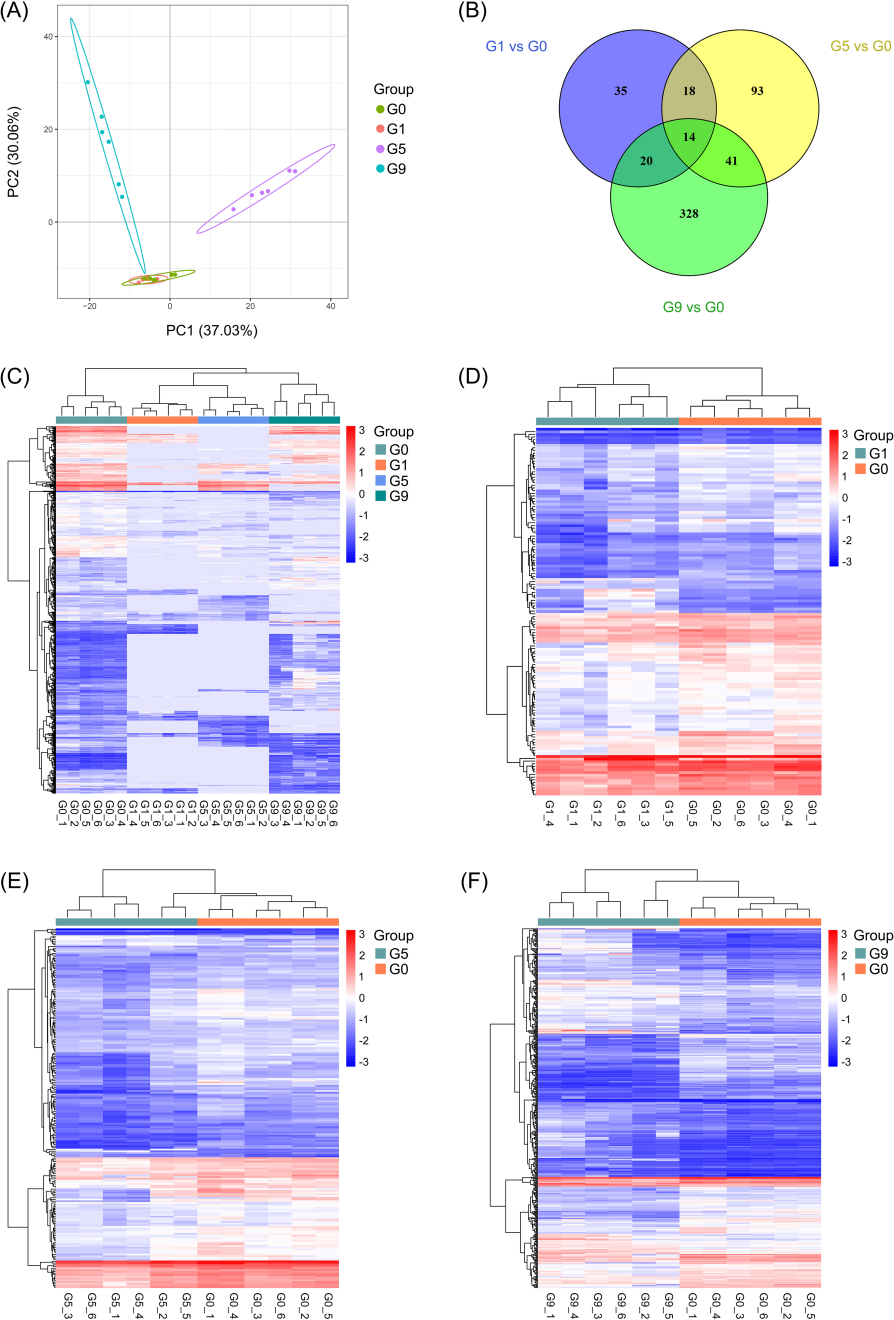
Metabolic profiling results of the DSS and DCS. **(A)** PLS-DA score plot. **(B)** Pairwise compare the DRMs in groups on the Venn diagram. **(C–F)** Hierarchical clustering analysis for the metabolites between different groups.

Different rhizosphere metabolites (DRMs) between DSS and DCS were analyzed in three comparison groups, G1 vs. G0, G5 vs. G0, and G9 vs. G0, in an effort to find new biopesticide to control TFW. There was little difference in the metabolites between the G0 and G1 groups, according to partial least squares discrimination analysis (PLS-DA), which showed a significant separation of metabolites between the G0 group and other groups with the exception of the G1 group (**Figure 1A**). **Figures 1C–F** displays the expression and distribution of DRMs among the four groups (**Supplementary Table S1**). From the G1 vs. G0, G5 vs. G0, and G9 vs. G0 comparison groups, a total of 87, 166, and 403 DRMs were obtained; 14 DRMs were the overlapping metabolites (**Figure 1B**; **Supplementary Table S2**). These 14 DRMs were choline bitartrate, N-acetyl-L-leucine, 4-hydroxyisoleucine, 1-(4-bromophenyl)-2-phenylethan-1-one, syringic acid, pantothenic acid, 3-(4-fluorophenoxy)-1-(1,4-thiazinan-4-yl)propan-1-one, L-adrenaline, DL-stachydrine, pyridoxine O-glucoside, acetaminophen glucuronide, linustatin, neosaxitoxin, and N-acetyl-L-glutamine, all of which were significantly downregulated in three comparison groups.

**Figure 2:**
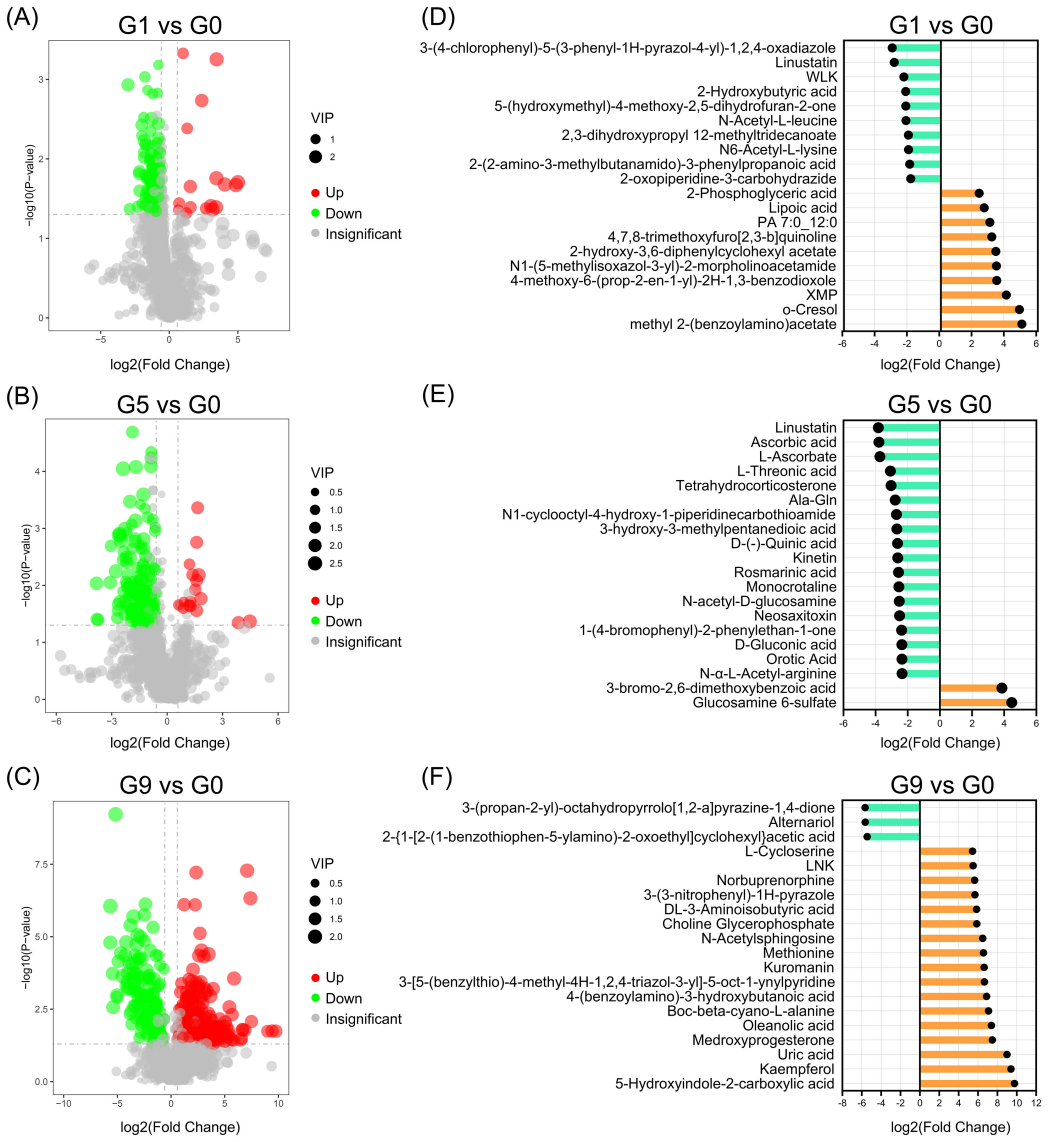
Up- and down-regulated DRMs in various groups. **(A–C)**. Volcano plots show the important discriminatory metabolites. **(D–F)**. The top twenty up- and down-regulated DRMs.

As illustrated in **Figures 2A–C**, G1, G5 and G9 exhibited significantly lower metabolites (72, 152, and 170) and significantly higher metabolites (15, 14, and 233) in comparison to G0. The top 20 DRMs in each of the three comparison groups were determined by ranking the absolute values of log2(fold change) (**Figures 2D–F**; **Supplementary Table S2**). In G1 vs. G0 group, the number of up- and down-regulated DRMs in the top 20 DRMs is equal, while the G5 vs. G0 and G9 vs. G0 groups had an uneven and opposite trend in up- and down-regulated DRMs.

**Figure 3:**
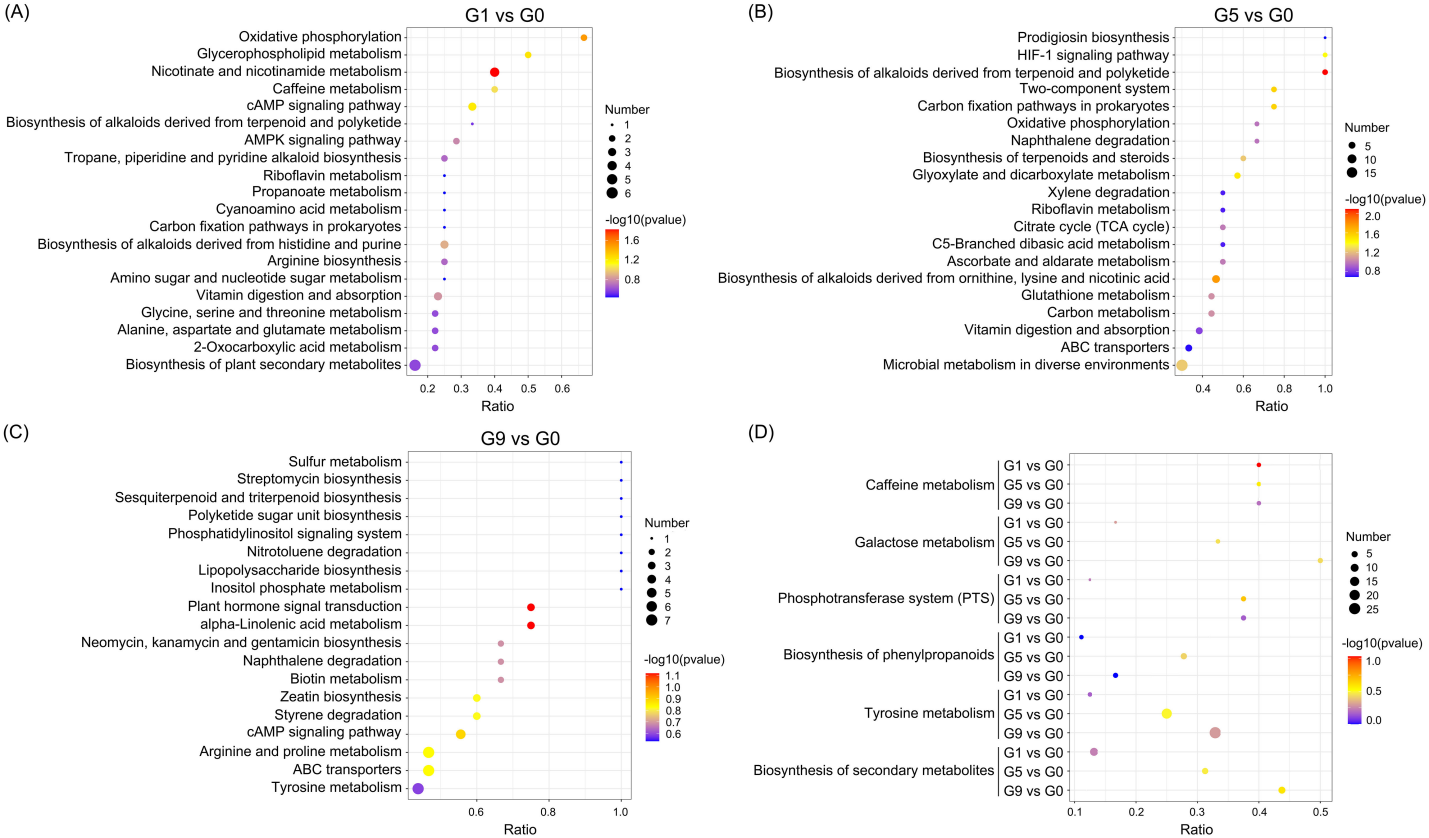
KEGG pathways enriched for DRMs in different comparison groups. **(A–C)**. The scatter plots of the enrichment statistics of the KEGG pathway in G1 vs. G0, G5 vs. G0 and G9 vs. G0, respectively. **(D)** The common KEGG pathway in three comparison groups.

Analyzing the KEGG annotation of metabolites allowed for a deeper understanding of the variations in metabolic pathways that DRM engaged in amongst the comparison groups (**Figure 3**; **Supplementary Table S3**). Nicotinic acid, NAD^+^, fumaric acid, and nicotinuric acid are the four metabolites that are significantly enriched in oxidative phosphorylation and nicotinate and nicotinamide metabolism in the G1 vs. G0 group (**Figure 3A**). The HIF-1 signaling pathway, carbon fixation pathways in prokaryotes, two-component systems, glyoxylate and dicarboxylate metabolism, biosynthesis of alkaloids derived from terpenoid and polyketide, biosynthesis of alkaloids derived from ornithine, lysine and nicotinic acid were all significantly enriched in the G5 compared to the G0 group (**Figure 3B**). The metabolic pathways that were most enriched in the G9 group as compared to the G0 group were tyrosine metabolism, ABC transporters, and arginine and proline metabolism (**Figure 3C**). The three comparison groups shared common metabolic pathways, including phosphotransferase system (PTS), tyrosine metabolism, galactose metabolism, biosynthesis of secondary metabolites, biosynthesis of phenylpropanoids, and caffeine metabolism (**Figure 3D**).

### 3.2 Correlation analysis

We investigated the relationship between TFW severity and DRMs content in order to pinpoint the specific DRMs that increased in DSS and decreased in DCS. We concentrated on DRMs with correlation coefficients (Corr coef) smaller than -0.7 in the G5 and G9 groups because the G1 group had less change in DRMs and milder disease symptoms. As shown in **Table 1** and **Supplementary Table S4**, 29 DRMs were obtained, among which 3-(4-fluorophenoxy)-1-(1,4-thiazinan-4-yl)propan-1-one, alternariol, 2-Hydroxyphenylalanine, dibenzoyl thiamine, 3-[4-methyl-1-(2-methylpropanoyl)-3-oxocyclohexyl]butanoic acid, 4-(cyclohexylmethyl)-6-(2-thienyl)-2,3-dihydropyridazin-3-one hydrate and phenazine-2-carboxylic acid were significantly negatively correlated with TFW severity (Corr coef >0.8). In addition, pantothenic acid, 3-(4-fluorophenoxy)-1-(1,4-thiazinan-4-yl)propan-1-one, acetaminophen glucuronide and linustatin were all severely decreased in three comparison groups.

**Table 1:** Correlation analysis between TFW severity and DRMs.

Name	G1 vs. G0			G5 vs. G0			G9 vs. G0			Correlation	
	log_2_(FC)	*p* value	VIP	log_2_(FC)	*P* value	VIP	log_2_(FC)	*P* value	VIP	Corr coef	*P* value
3-(4-fluorophenoxy)-1-(1,4-thiazinan-4-yl)propan-1-one	-1.25	0.01	1.15	-2.23	3.25E-03	2.01	-3.42	2.57E-04	2.11	-0.87	2.81E-08
Alternariol	-0.26	0.60	0.12	-1.91	2.28E-03	1.27	-5.65	8.81E-07	2.20	-0.87	4.30E-08
2-Hydroxyphenylalanine	-0.72	0.02	0.45	-1.820964	7.52E-02	1.68	-3.10	2.06E-03	1.52	-0.87	4.3E-08
Dibenzoyl Thiamine	-0.86	0.11	0.72	-1.57	0.02	1.43	-5.16	5.97E-10	2.15	-0.83	4.00E-07
3-[4-methyl-1-(2-methylpropanoyl)-3-oxocyclohexyl]butanoic acid	-0.77	0.04	0.80	-1.17	0.03	1.31	-3.49	2.03E-06	2.21	-0.83	5.54E-07
4-(cyclohexylmethyl)-6-(2-thienyl)-2,3-dihydropyridazin-3-one hydrate	-0.79	0.13	0.75	-2.19	3.98E-03	1.97	-3.29	7.78E-05	1.90	-0.82	1.03E-06
Phenazine-2-carboxylic acid	-0.81	0.03	0.57	-0.79	3.55E-02	0.58	-4.50	2.32E-04	2.18	-0.81	1.38E-06
2-(Formylamino)Benzoic Acid	-0.30	0.69	0.85	-1.49	5.55E-02	1.23	-2.80	2.64E-03	1.83	-0.79	4.11E-06
6,6-dimethyl-4-piperidino-5,6-dihydro-2H-thiine-2-thione	-0.73	0.16	0.67	-2.17	1.19E-03	1.33	-3.64	3.68E-03	1.93	-0.79	5.30E-06
Phenylpropiolic acid	-1.16	0.06	0.95	-1.94	0.03	1.85	-3.27	1.91E-04	1.75	-0.79	5.30E-06
Ouabain	-0.87	0.25	0.82	-1.93	0.03	1.39	-4.15	4.93E-04	1.99	-0.78	8.61E-06
Rosmarinic acid	-0.76	0.14	0.99	-2.58	1.23E-03	2.19	-3.53	1.79E-04	1.84	-0.77	1.09E-05
Linustatin	-2.89	0.04	1.58	-3.83	0.01	2.17	-5.40	2.70E-03	1.92	-0.76	1.36E-05
N-Acetyl-Asp-Glu	-0.94	0.23	0.90	-2.22	0.01	1.80	-3.19	7.68E-04	1.81	-0.76	1.70E-05
(±)12(13)-DiHOME	0.01	0.94	0.31	-1.28	1.55E-03	2.30	-0.99	3.89E-03	1.22	-0.75	2.11E-05
4-amino-2-(2,4-dimethoxyanilino)-5-pyrimidinecarbonitrile	-0.72	0.22	0.73	-1.95	0.01	1.55	-4.28	4.41E-05	1.92	-0.75	2.11E-05
4-Methylhippuric acid	-0.03	0.98	0.91	-0.97	5.22E-02	1.35	-1.74	3.55E-03	1.66	-0.75	2.61E-05
Benzyl 6-O-beta-D-glucopyranosyl-beta-D-glucopyranoside	-1.59	0.00	1.13	-0.11	1.84E-01	1.05	-4.18	1.56E-05	2.05	-0.74	3.20E-05
N-(2,5-diethoxy-4-morpholinophenyl)-4-methoxybenzenesulfonamide	-0.76	0.05	0.80	-0.69	9.66E-02	1.39	-2.85	1.01E-05	1.89	-0.74	3.20E-05
Benzo[E]pyrazolo[5,1-c][1,2,4]triazin-8-yl N,N-dimethylcarbamate	-0.79	0.11	1.19	-1.88	2.04E-05	1.85	-1.92	9.79E-03	1.66	-0.74	3.91E-05
Estrone sulfate	-0.93	0.17	0.92	-2.14	0.01	1.60	-3.90	7.84E-05	1.87	-0.74	3.91E-05
5-acetyl-2,6-dimethyl-1,2,3,4-tetrahydropyridin-4-one	-1.21	0.00	1.26	-0.78	6.61E-02	1.80	-2.47	1.77E-06	1.81	-0.74	3.91E-05
Scopoletin	-1.20	0.06	0.99	-1.90	0.03	1.64	-3.33	2.41E-03	1.71	-0.73	4.75E-05
D-(+)-Glucose	0.36	0.60	0.44	-1.41	0.04	1.28	-3.30	1.79E-04	1.65	-0.73	4.75E-05
Acetaminophen glucuronide	-1.53	0.04	1.11	-2.00	0.01	2.01	-4.04	1.86E-04	1.79	-0.73	5.76E-05
Dl-3-Hydroxy-kynurenine	-0.80	0.10	0.79	-0.95	5.38E-02	1.12	-4.02	6.30E-04	1.99	-0.73	5.76E-05
Pantothenic acid	-1.27	0.02	1.50	-1.39	0.02	1.71	-2.30	1.02E-03	1.78	-0.72	8.33E-05
3-hydroxy-3-methylpentanedioic acid	-0.79	0.27	1.36	-2.68	0.03	1.50	-3.81	4.61E-03	1.67	-0.72	8.33E-05
1,4-dihydroxy-1,4-dimethyl-7-(propan-2-ylidene)-decahydroazulen-6-one	-1.29	0.01	1.32	-0.47	1.79E-01	0.99	-3.13	8.81E-06	2.05	-0.71	1.19E-04

FC, fold change; VIP, variable importance in projection; Corr coef, correlation coefficient.

### 3.3 Screening of DRMs with anti-*F. oxysporum* effect

**Figure 4:**
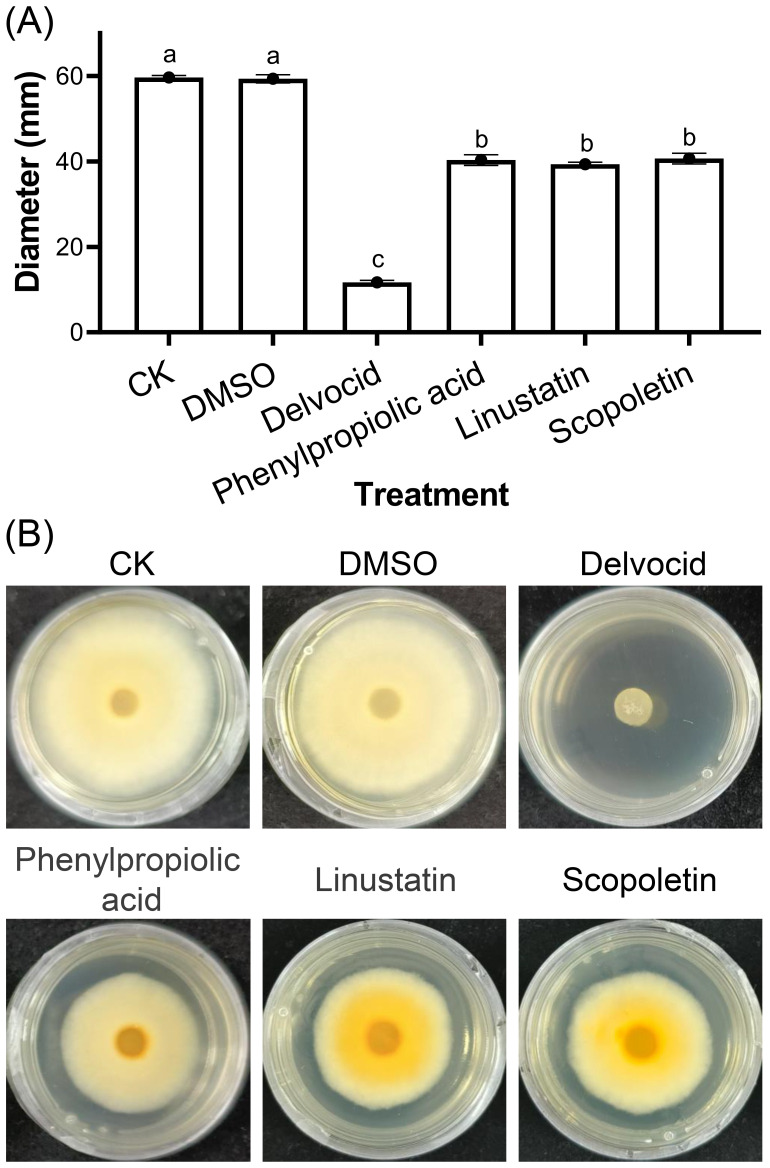
Effects of 0.5 mM DRMs on *F oxysporum* physiology **(A)** and representative images **(B)**. DMSO and 0.2 g·L^-1^ Delvocid were used as negative and positive control, respectively. Different lowercase letters indicate significant difference (*P*< 0.05) according to Duncan’s multiple range test. The number of biological replicates for each experiment was n=3.

**Figure 5:**
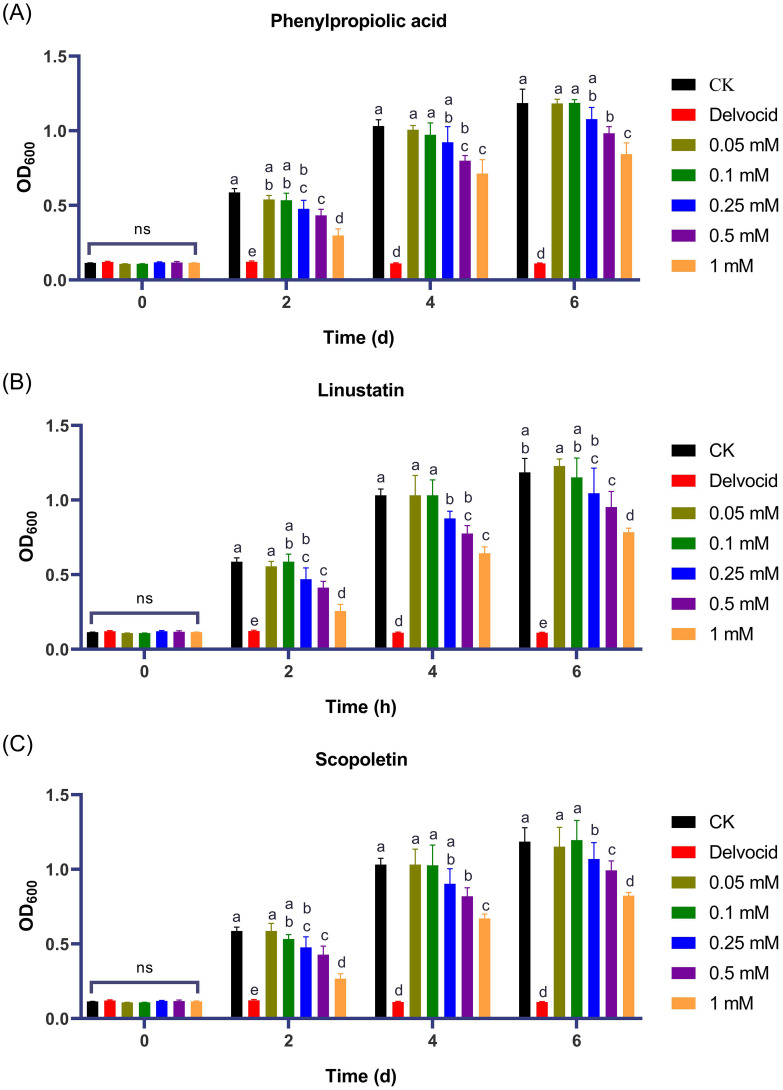
Effects of phenylpropiolic acid **(A)**, linustatin **(B)** and scopoletin **(C)** on the growth of *F oxysporum*. Delvocid (0.3 g·L^-1^) were used as positive control. Different lowercase letters indicate significant difference (*P*< 0.05) according to Duncan’s multiple range test; ns, not significant. The number of biological replicates for each experiment was n=3.

To investigate the activities of DRMs that are negatively correlated with TFW severity on the physiology and growth of *F. oxysporum*, 18 DRMs of alternariol, 2-hydroxyphenylalanine, dibenzoyl thiamine, phenazine-2-carboxylic acid, 2-(formylamino)benzoic acid, phenylpropiolic acid, ouabain, rosmarinic acid, linustatin, N-acetyl-Asp-Glu, 4-Methylhippuric acid, estrone sulfate, scopoletin, D-(+)-glucose, acetaminophen glucuronide, Dl-3-hydroxy-kynurenine, pantothenic acid, and 3-hydroxy-3-methylpentanedioic acid from **Table 1** that we could purchase on the market were tested using the plate inhibition method. The result showed that only phenylpropiolic acid, linustatin and scopoletin showed inhibitory effect against *F. oxysporum* at concentrations of 0.5 mM after 6 d co-culture (**Figure 4**). To further dissect the inhibitory effect of these three DRMs on *F. oxysporum*, we examined the effects of different concentrations of three DRMs on the spore germination and spore growth of *F. oxysporum*. **Figure 5** showed that phenylpropiolic acid, linustatin and scopoletin all significantly inhibited the growth of *F. oxysporum* at concentrations of 0.25 mM after 2 d, and only 0.25 mM linustatin showed strong inhibitory effects both 2 d and 4 d. At concentrations of 0.5 mM or above, the three DRMs exhibited strong inhibitory effects on *F. oxysporum* at all times tested.

### 3.4 Chemotaxis of DRMs to *F. oxysporum*

**Figure 6:**
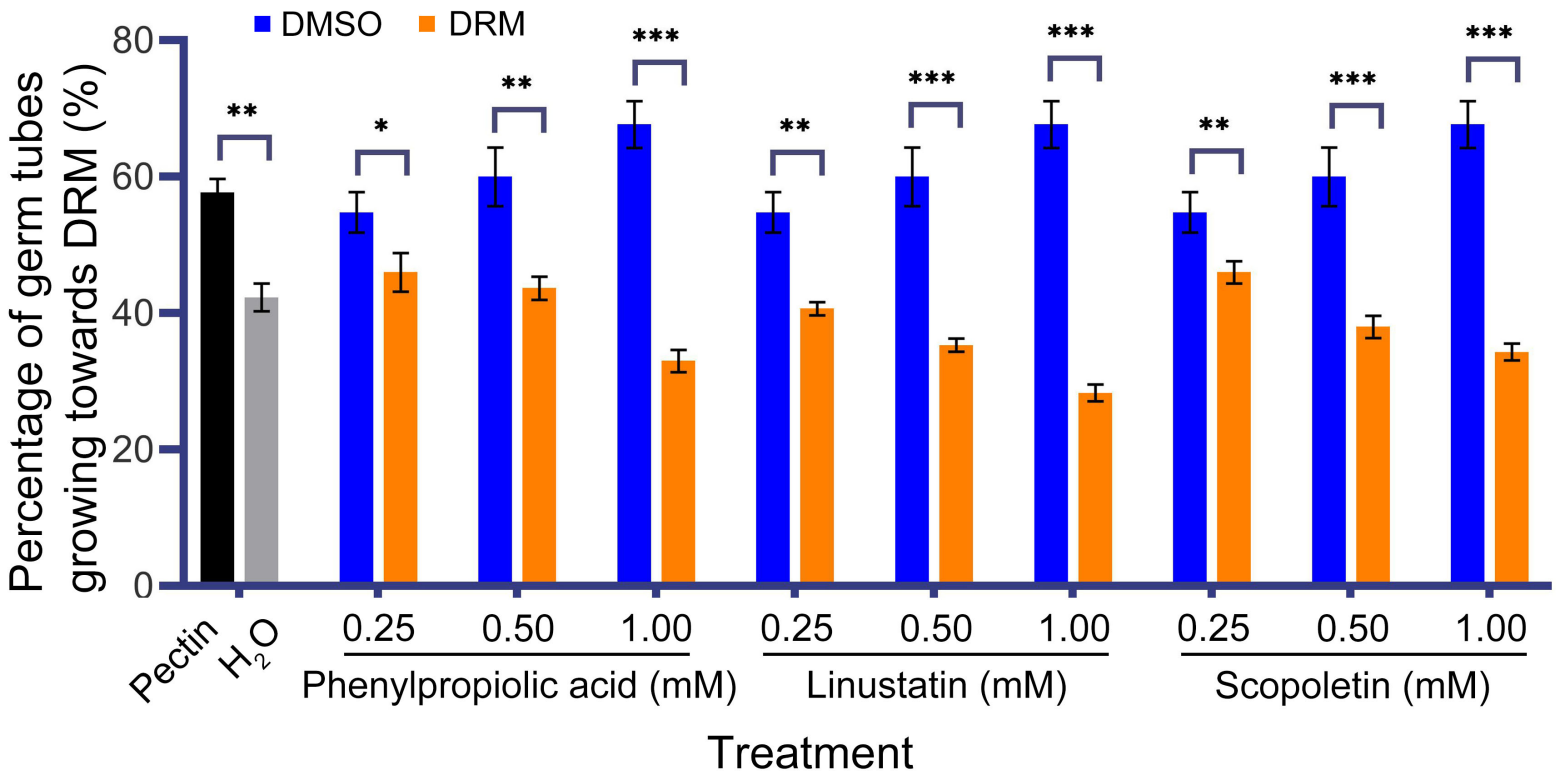
Chemotactic effect of *F. oxysporum* to DRMs. 1% Pectin was used as a positive control. Asterisks indicate significant differences between DRMs and its solvent: *P< 0.05, **P< 0.01, ***P< 0.001, student’s t test.

To investigate the effect of three DRMs on hyphal growth of *F. oxysporum*, we performed a chemotaxis assay. Our results showed that *F. oxysporum* could be significantly attracted by the positive control pectin, while it showed phobotaxis effect toward phenylpropiolic acid, linustatin and scopoletin at all three concentrations. In particular, linustatin and scopoletin had highly significant detrimental effects on fungal hyphae even at a low concentration of 0.25 mM (**Figure 6**).

### 3.5 Effects of DRMs on the integrity and permeability of the mycelial cell membrane of *F. oxysporum*

**Figure 7:**
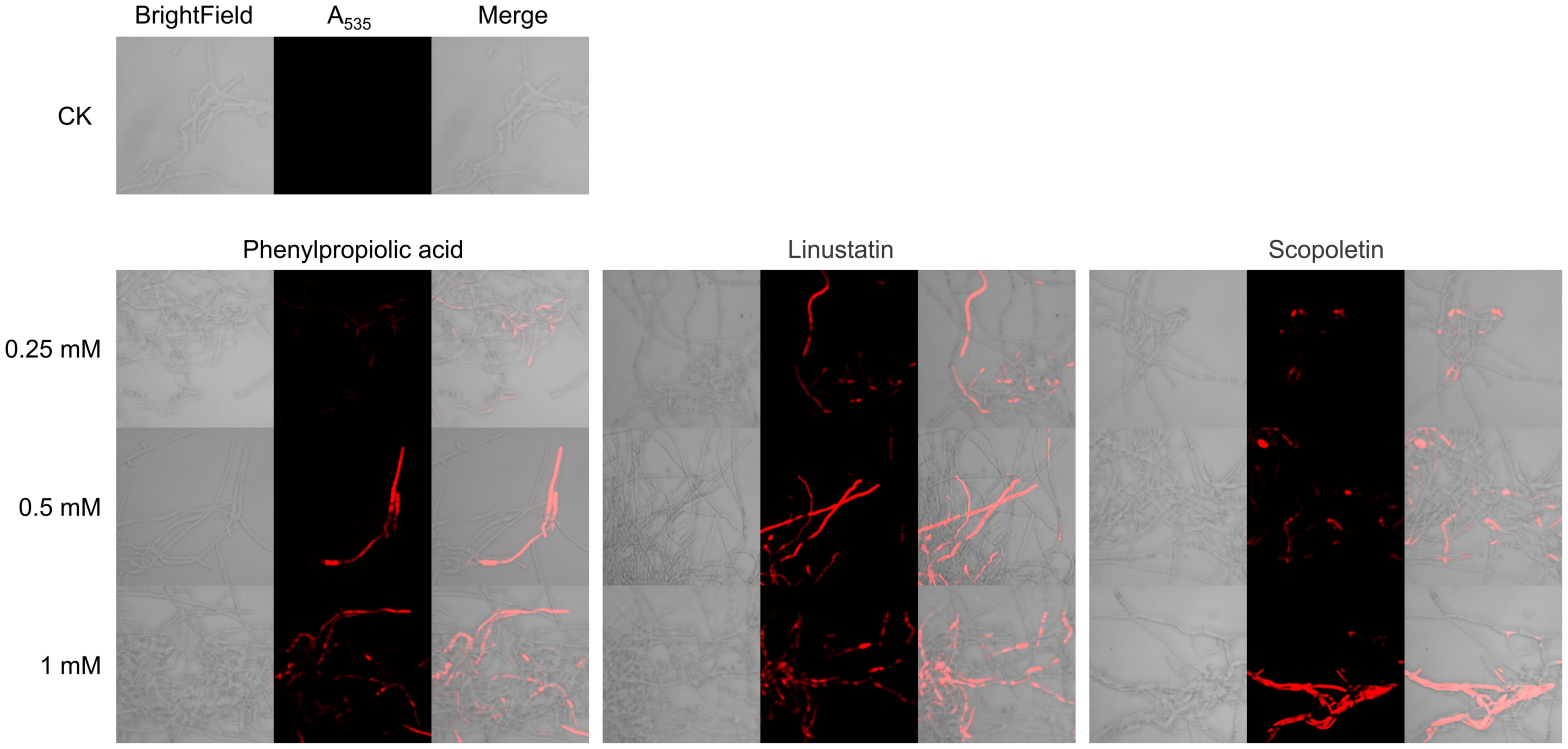
PI staining observation of *F. oxysporum* mycelia treated with DRMs.

We examined the effects of DRMs on cell membrane integrity of *F. oxysporum* using PI staining. Microscopic observations showed that hyphae of the untreated control were barely stained with PI, whereas cells treated with phenylpropiolic acid, linustatin, and scopoletin all emitted red fluorescence, indicating extensive cell death and membrane permeation (**Figure 7**). Furthermore, as the DRM dose increases, the proportion of PI-stained cells in the total number of cells gradually increases in a dose-dependent manner.

**Figure 8:**
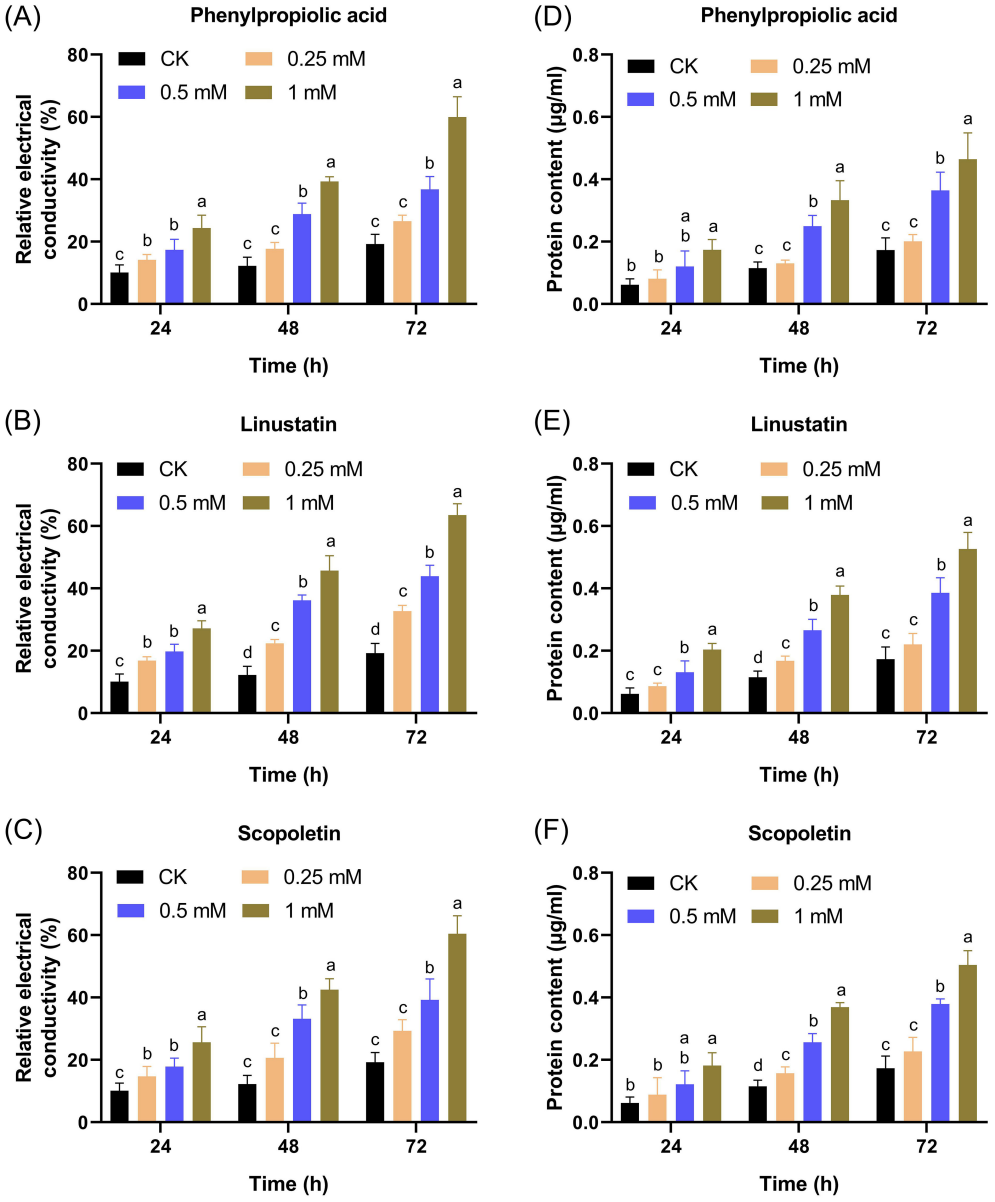
Effects of different concentrations of DRMs on the extracellular relative conductivity **(A–C)** and extracellular protein content **(D–F)** of *F. oxysporum*. Different lowercase letters indicate significant difference (*P*< 0.05) according to Duncan’s multiple range test; ns, not significant. The number of biological replicates for each experiment was n=3.

To further confirm the damaging effect of DRMs on the cell membrane, we measured the REC and protein content changes in the supernatant after treatment with DRMs. The REC of all treated groups as well as the CK group increased over time and in a dose-dependent manner (**Figures 8A–C**). The groups treated with the three DRMs at a concentration of 0.5 mM or higher showed a significant increase in REC compared to the CK group at all times tested, and only linustatin increased the REC of *F. oxysporum* at concentrations as low as 0.25 mM. In addition, the REC of the linustatin-treated group was much higher than that of the other two groups after 24, 48, and 72 h of incubation, indicating that linustatin had a stronger damaging effect on the cell membranes of *F. oxysporum* than the other two DRMs. As shown in **Figures 8D–F**, the extracellular protein content of *F. oxysporum* also increased significantly after treatment with three DRMs above 0.25 mM for 24 h, 48 h, and 72 h. These results collectively suggest that the three DRMs inhibited *F. oxysporum* by disrupting the cell membrane and increasing the membrane permeability of *F. oxysporum*.

## 4 Discussion

*F. oxysporum* is a soil-borne pathogen that poses a serious threat to the cultivation of hundreds of crops worldwide. Numerous studies have shown that plant roots and beneficial microorganisms residing in the rhizosphere secrete a variety of secondary metabolites to combat soil-borne pathogens (Wu, 2008; Mazurier et al., 2009; Yuan et al., 2012; Wang et al., 2013a; Yan et al., 2023). Therefore, it is an effective, environmentally friendly, and safe method to control soil-borne disease by studying and applying anti-microbial metabolites from root exudates and probiotics present in the rhizosphere. In the present study, we discovered three natural metabolites with antifungal activity through comparative metabolomic analysis of DSS and DCS, of which phenylpropiolic acid and linustatin were reported for the first time. These three metabolites all have potent inhibitory effects on the spore germination and mycelium growth of *F. oxysporum.*

Through metabolomic analysis of DSS and DCS, we found that the relative contents of 298 DRMs, including phenylpropiolic acid, linustatin and scopoletin, were greatly reduced in DCS compared to DSS. These down-regulated metabolites were mainly phenolic acids, phenylpropanes, dipeptides and glycosides, which were enriched in metabolic pathways such as phenylpropanoids biosynthesis, tyrosine metabolism, and caffeine metabolism. The phenylpropanoid pathway is a classic metabolic pathway associated with plant growth and stress responses that can inhibit or eliminate ROS in cells under biotic or abiotic stress through the synthesis of antioxidants such as phenols and flavonoids, thereby protecting DNA, proteins, membrane lipids and other organ components from severe damage (Fu et al., 2023). On the other hand, the lignin synthesized in this pathway can promote cell lignification, thicken the cell wall and form a physical barrier to prevent the invasion of pathogens and insects (Lu et al., 2022). In the present study, the content of fumaric acid, scopoletin, L-phenylalanine and citric acid, which are involved in the biosynthesis of phenylpropanoids, deceased in DCS compared to DSS, suggesting that these metabolites were depleted to protect the plants from infection with *F. oxysporum*. This result was consistent with the previous study by Fu et al. (2023), in which phenylalanine, caffeic acid, trans-ferulic acid, and mustard acid, which are associated with phenylpropanoid biosynthesis of ramie growth were significantly decreased in the obstacle group compared to the CK group.

In the subsequent correlation analysis and antifungal experiment, it was confirmed that scopoletin had an inhibitory effect on *F. oxysporum*. Scopoletin is a type of coumarin phytoalexin that is widely distributed throughout the plant kingdom (Gnonlonfin et al., 2012). Scopoletin is known to have antibacterial and antifungal effects. Scopoletin isolated from *Ixora javanica* and *Greenea montana* was reported to have inhibitory activity against *Enterococcus faecium* UCLA 192 with a minimum inhibitory concentration (MIC) value of 128 μg/mL (Buathong et al., 2019). Stringlis et al. (2018) reported that scopoletin exhibits a selective inhibitory effect on the soil-borne fungal pathogens *F. oxysporum* and *Verticillium dahlia*. In addition, they found that scopoletin not only reduced the growth of *F. oxysporum* and deterred the fungal hyphae, but also hindered the germination of *F. oxysporum* spores and inhibited the pigmentation of *F. oxysporum* mycelium, which is consistent with our results.

In addition to scopoletin, two other DRMs phenylpropiolic acid and linustatin with anti*-F. oxysporum* activity were also picked out. Phenylpropiolic acid was found to be a potent inhibitor of *Sporosarcina pasteurii* urease with an inhibition constant of 37.1 μM (Macegoniuk et al., 2017), and also possessed antibacterial activity against *Brucella abortus* 544 with an MIC value of 250 μg/mL (Kazemizadeh et al., 2016). However, the antifungal effect of phenylpropiolic acid has not yet been documented. This study reports for the first time that phenylpropiolic acid has an inhibitory effect on *F. oxysporum*, expanding the function and application potential of phenylpropiolic acid.

Linustatin is a cyanogenic glycoside that occurs in many plants as a phytoanticipin (Sohail et al., 2023). Cyanogenic glycoside exerts its defense function by releasing toxic free hydrogen cyanide (HCN) to aerobic organisms as an inhibitor of respiration and metal containing enzymes (Cressey and Reeve, 2019). Some studies depicted that high level of linustatin in foods threaten the health of human and herbivores, but little is known about its antimicrobial effects (Schrenk et al., 2019; Zuk et al., 2020). The present study addresses this limitation that linustatin has suppressive activity against *F. oxysporum*.

The cell membrane is a microbial barrier that can block the entry of external substances and prevent the outflow of internal substances. When the cell membrane is damaged, membrane fluidity is inhibited, membrane permeability changes, and intracellular electrolyte leakage occurs, eventually affecting microbial growth and leading to cell death (Di Pasqua et al., 2007). Our results showed that scopoletin, phenylpropiolic acid and linustatin could all inhibit *F. oxysporum* by impairing cell membrane integrity and increasing cell membrane permeability of *F. oxysporum* mycelium, which was similar to studies in which osthole and ferulic acid exerted antifungal activity by disrupting cell membrane integrity and dynamic equilibrium of *F. oxysporum* (Hu et al., 2023; Yan et al., 2023). In this study, the antifungal mechanism of three DRMs was discussed from the perspective of cell membrane integrity and permeability. It is necessary to comprehensively clarify the antifungal mechanisms from other aspects, such as the effect on *F. oxysporum* gene expression and induction of tobacco disease resistance. Furthermore, the application effects of these three DRMs still need to be further evaluated in the field.

## 5 Conclusions

This aim of this study was to discover natural metabolites with inhibitory effects of *F. oxysporum* from the tobacco rhizosphere for the control of TFW. Phenylpropiolic acid, linustatin and scopoletin were identified and confirmed to have suppressive activity against *F. oxysporum* through comparative metabolomics, correlation analysis and antifungal experiments. Furthermore, the antifungal mechanism of three DRMS by disrupting cell membrane integrity and permeability of *F. oxysporum* was preliminarily investigated.

## Data Availability

The original contributions presented in the study are included in the article/**Supplementary Material**. Further inquiries can be directed to the corresponding authors.
